# Value of strain-wave sonoelastography as an imaging modality in assessment of benign acute myositis in children

**DOI:** 10.3906/sag-2103-290

**Published:** 2021-08-07

**Authors:** Ayşe Seçil EKŞİOĞLU, Ayla AKCA ÇAĞLAR, Seda KAYNAK ŞAHAP, Can Demir KARACAN, Nilden TUYGUN

**Affiliations:** 1 Division of Pediatric Radiology, Department of Radiology, Faculty of Medicine, Yıldırım Beyazıt University, Ankara, Turkey; 2 Division of Pediatric Emergency Care, Department of Pediatrics, Dr Sami Ulus Obstetrics and Gynecology, Pediatric Health and Disease Training and Research Hospital, University of Health Sciences, Ankara, Turkey; 3 Division of Pediatric Radiology, Department of Radiology, Pediatric Radiology, Dr Sami Ulus Obstetrics and Gynecology, Pediatric Health and Disease Training and Research Hospital, University of Health Sciences, Ankara, Turkey

**Keywords:** Sonoelastography, myositis, children

## Abstract

**Background/aim:**

Although sonoelastography is applied in assessment of many organs, studies for evaluation of muscles are very few in number and are mostly limited to adults. With this prospective study, we aimed to evaluate the value of sonoelastography in assessment of influenza related benign acute myositis in children.

**Materials and methods:**

This study enrolled 25 patients with a clinical diagnosis of benign acute childhood myositis (BACM) and 25 age and sex-matched healthy controls. All patients presented to our emergency department with the complaint of inability to walk and had increased serum creatine kinase (CK) levels. All patients underwent strain elastography of the gastrocnemius muscle, and an elastography score was assigned to each patient by using a previously published 5 point-color scoring system. The findings were compared with those of the control group.

**Results:**

No statistically significant difference was detected regarding age, weight, height, or body mass index (BMI) between patient and control groups. A statistically significant difference was found between the final elastography scores of the patient and control groups, mean values being 4.16 ± 0.75 versus 3.08 ±0.40, respectively (p < 0.001). Sonoelastography yielded a sensitivity of 80%, positive predictive value of 87%, specificity 88%, negative predictive value of 81.5%, and an overall accuracy of 84 %.

**Conclusion:**

Sonoelastography proves to be a valuable tool for diagnosis of BACM. It is one of the available ultrasound techniques in a radiology department and may particularly evolve to become a useful routine ancillary technique for investigation and follow-up in these cases.

## 1. Introduction

Benign acute childhood myositis (BACM) is a rare, muscle disorder characterized by sudden onset of calf pain with an isolated finding of elevated serum creatine kinase (CK), being preceded by an influenza-like illness. Although many causes of upper respiratory tract infection like respiratory syncytial virus or adenoviruses may lead to myositis, the disorder most commonly occurs due to influenza viruses. Thus, its incidence increases during influenza epidemics in winter. It predominantly affects school-aged children, with a predilection to boys. Whether it is caused by direct viral invasion or through an immunological route is not clear [1].

Although it is a self-limiting condition, it is very alarming for the family to see that a previously healthy child is suddenly not able to walk after a minor viral infection. These families usually come to the emergency department after the onset of myositis symptomatology with concerns about a cerebrovascular event. If the child is not verbally communicative yet, the concerns can even escalate more. It’s also important to make its differential diagnosis from Guillain–Barré syndrome and more severe disorders that cause myoglobinuria. In BACM, muscle biopsy frequently yields nonspecific findings, and, thus, it is unnecessary. EMG is not carried out in routine patient management unless there is strong suspicion for a primary muscle disease. Most of the time there is need to validate the diagnosis with imaging.

As an imaging modality to support the diagnosis MR is not always approachable. Findings on gray scale ultrasound can be nonspecific [2,3]. Advanced ultrasound techniques can enhance the diagnostic sensitivity of conventional gray-scale imaging [4].

At this point sonoelastography, which is one of the latest advances in ultrasonography and offers a good functional and quantitative evaluation by measuring the stiffness / elasticity of the muscles, is promising as an accessible, low-cost, radiation-free technique [5].

Although since its introduction into clinical practice, sonoelastography has been applied in assessment of many lesions from thyroid nodules to liver pathologies [6]; studies for evaluation of muscles with sonoelastography are very few in number and are mostly limited to adults [7–11].

While there is a limited number of reports in the literature describing abnormal muscle strain in children with spastic cerebral palsy and juvenile idiopathic inflammatory myopathies [12–14], to date, we could come across only one very recent study with a limited number of patients, assessing muscle elasticity in children with acute myositis [15].

We, therefore, undertook a prospective study with a larger group of patients to determine the value of strain elastography as an imaging modality for the assessment of children diagnosed with BACM.

## 2. Materials and methods

Informed consent was obtained from the parents of the patients and controls, and the study was approved by institutional Research Ethics Committee. During the conduct of this study, all relevant ethical safeguards have been met in accordance with the ethical standards of the World Medical Association (Declaration of Helsinki).

Initially, 28 consecutive patients (22 boys, 6 girls) admitted to our hospital’s pediatric emergency department between April 2019 and April 2020 and received the diagnosis of BACM depending on a combination of patient’s history, clinical assessment and laboratory results were included in the study. All patients had flu-like symptoms starting within the last 7 days before admission to the hospital. They also had sudden onset of bilateral calve pain and/or inability to walk accompanied by elevated serum CK levels. Physical examination revealed no abnormal neurological findings. Exclusion criteria were established as family or past history of muscular or rheumatic disease or any systemic, neurological or musculoskeletal disorder, or history of long-term medication use or trauma. Three patients (2 boys and 1 girl) were excluded from the study: one for having a history of rheumatic disease, one for having a family history of musculoskeletal disorder and one for having a history of previous trauma. The remaining 25 patients consisted of 20 boys and 5 girls with an age range of 4.30–15.08 and a mean age of 8.03 ± 2.84 years. The control group consisted of 25 individually age and sex matched subjects (20 boys, 5 girls, age range 4.04–15.24, mean age 8.11 ± 3.01years) admitted to the outpatient clinics of our hospital without any specific musculoskeletal complaint or active infection history in the last 15 days and referred to the ultrasonography unit of our radiology department for illnesses such as enuresis, hydrocele, liver hemangioma, etc. or were just healthy siblings of the patients.

All the subjects were examined using a Toshiba Aplio 500 (Toshiba Medical System Corporation, Tokyo, Japan) ultrasound machine equipped with compression–strain elastography using an L 12-5 linear-array transducer. After 15 min of restricted activity period muscle structures of the calves of all subjects were scanned in prone position with legs in extension and ankles in neutral position. Strain elastography was performed by the same experienced pediatric radiologist at one sitting (ASE) using an L12-5 linear-array transducer. Ultrasound gain, depth, focal points, and transducer frequency settings were kept constant in all image scans. Sonoelastography was carried out in real time, using sagittal sections of the midportion of the calve muscles symmetrically from each side, by using an L12-5 linear-array transducer oriented longitudinally to the muscle fibers with 90° to the skin. The adequacy of the manual compression applied, could be checked by a real time compression feed-back bar. A color map representing tissue elasticity was superimposed on the gray-scale sonographic image, with red indicating the most elastic tissues, green indicating tissues with intermediate elasticity, and blue indicating the least elastic tissues. Images obtained from both the right and left sides were stored in the archive.

**Figure 1:**
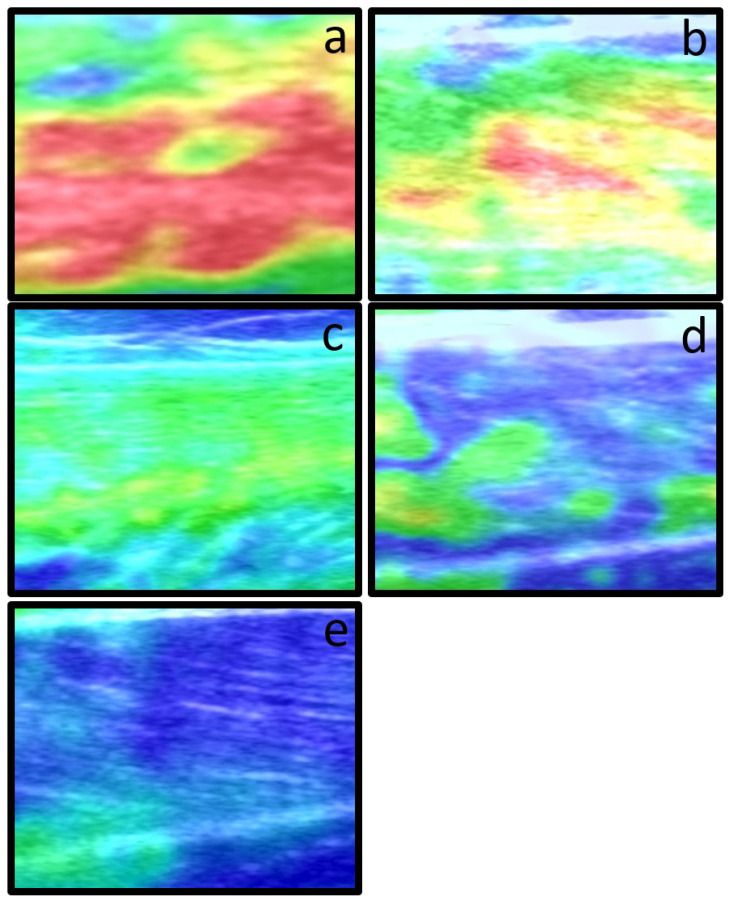
Elastography color grading scale. A five-point color grading scale was used for assessing muscle stiffness with elastography. (a) Score 1 indicates greatest elasticity (softest muscle), (b) score 2, and (c) score 3 are examples for intermediate muscle elasticity at rest, (d) score 4 and (e) score 5 indicate lowest elasticity (hardest muscles) generally seen in patients with myositis.

**Figure 2:**
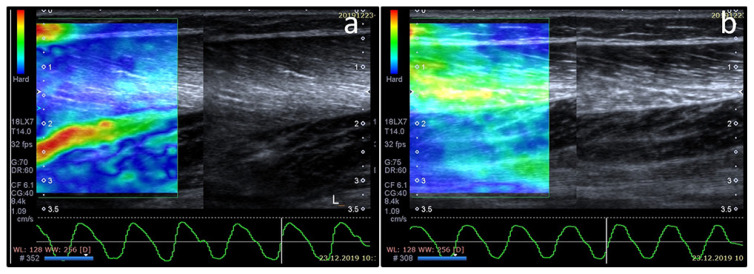
8-year-old male with inability to walk, flu-like symptoms, and increased serum CK level. Strain elastography of the symptomatic right side reveals an elastography score of 5 (a), while contralateral side has a score of 3 (b). Also note the increase in muscle thickness and echogenicity on the gray scale ultrasound on the symptomatic right side when compared to contralateral.

Elastography data of the patients and controls were analyzed separately by two pediatric radiologists (ASE and SKS) blinded to the clinical diagnosis of the subjects. For elastographic evaluation the predominant color, the presence of significant amounts of secondary color was assessed and an elastography score was assigned to each muscle by using a previously published 5 point-color scoring system [16] as summarized in Table 1 (Figures 1 a–1e). If different scores were assigned to the left and right sides of a subject the bigger score was accepted as the final score (Figures 2a, 2b). A score of 1 indicated greatest elasticity (softest muscle), score of 3 indicated medium elasticity, and a score of 5 indicated lowest elasticity (hardest muscle). Interobserver agreement for elastography scoring was calculated. A final reading was made jointly, and a score was decided for each patient in consensus.

SPSS version 21.0 (IBM Corp. Released 2012. Armonk, NY, USA) was used for statistical analysis. Descriptive statistics were presented as mean, standard deviation, median, minimum, maximum, frequency, and a percentage. Chi-square test was used to compare categorical variables. Independent samples t-test was used to compare continuous variables. Spearman correlation test was used to determine the linear association between variables. We calculated the sensitivity, specificity, and positive and negative predictive values for assessment of active disease by imaging modality using physician global assessment of disease activity as gold standard. A p value of <0.05 was considered as statistically significant.

## 3. Results

The demographic features of the participants are summarized in Table 2. No statistically significant difference was detected regarding age, weight, height, or body mass index (BMI) between patient and control groups.

A total of 92% of the patients had flu like symptoms for less than 5 days before admission, while 8% had symptoms for longer, between 5 and 7days. The time between the onset of the flu-like symptoms and calve pain was in the range of 1to 4 days with a mean value of 2 days. The duration of the musculoskeletal symptoms at the time of the examination was less than 24 h in 64% of the patients.

Nasal swab tests revealed positive antibodies for 33% of the children in the patient group for Influenza A or Influenza B. The test was negative in 52% for either. The serum CK levels of the patient group varied between 507 and 18196 with a mean value of 4325.24.

Sonoelastography scores of the patient group and controls are summarized in Table 3. Interobserver agreement for elastography score between radiologists was calculated yielding a good kappa value of 0.85. The final scores were reached in consensus. While no statistically significant difference could be detected between the elastography scores of right and left sides either in the patient or control groups (p < 0.05 for both), the final scores (the highest score obtained from right or left side) showed a statistically significant difference between the patient and control groups, mean values 4.16 ± 0.75 versus 3.08 ± 0.40, respectively (p < 0.001).

No positive correlation could be identified between the CK values and elastography scores at the time of diagnosis (rs = −0.328, p = 0.109). 4 patients with the lowest CK values (507, 691, 775, and 1194) had an elastography score of 5, whereas only one of the 8 patients with the highest CK levels had an elastography score of 5.

There is a weak to moderate negative correlation between the duration of the disease and elastography scores, which is not found to be statistically significant (rs= −0.186, p = 0.372). The elastography scores were higher if imaging was done within the first 3 days after the onset of musculoskeletal symptoms and tended to decrease after 3 days duration, but not in a strong linear fashion.

This is quite the opposite for the CK levels. There is a non-significant positive correlation between the CK levels and the duration of the symptoms (rs = 0.208, p = 0.318).

Using physician global assessment consisting of patient’s history, physical examination, laboratory tests as the gold standard for the diagnosis of acute benign myositis, and accepting an elastography score of 4 or 5 as decreased elasticity, sonoelastography had a sensitivity of 80 % and specificity of 88% for the diagnosis of BACM. The positive and negative predictive values for sonoelastography in the detection of acute myositis were 87% and 81.5%, respectively. Overall accuracy was 84%.

## 4. Discussion

Elastography, being one of the latest advances in ultrasound technology, mainly allows assessment of tissue elasticity. Two primary methods of performing elastography are strain elastography and shear-wave elastography. Strain elastography is also referred to as compression elastography and depends on the principle that harder tissues are less compressible and demonstrate lower strain, while softer tissues are more compressible and demonstrate higher strain. In strain-based elastography, force is applied by the application of probe pressure or through endogenous mechanical force (e.g., carotid pulsation) In shear-wave based elastography, a tissue shear-wave is induced by the imaging system [4–6].

Since its first introduction into clinical practice in 1990’s, ultrasound elastography has proved useful in the evaluation of breast and thyroid masses and pathological processes in internal organs like liver, spleen, kidney, pancreas, and prostate. However, its applications to musculoskeletal imaging emerged later [17–20]. MRI has been the method of choice in most musculoskeletal diseases; however, it is neither very accessible nor cost effective to carry out especially for every suspected BCAM case admitted to an emergency department. Also, sedation might be needed for younger children. Ultrasonography, being fast, low-cost, radiation-free and not requiring sedation is a tool usually easily accessible. Sonoelastography, being one of the advanced ultrasound techniques can enhance the diagnostic sensitivity of conventional gray-scale ultrasound.

Initially sonoelastography was found valuable in the evaluation of normal and pathological tendons [21–23]; however, studies for evaluation of muscles have not been fully developed and are mostly limited to adults [7–11]. To date, there has been only one recent study assessing muscle elasticity in acute myositis in a small group of 16 children [15].

In this prospective study, a larger group of 25 patients and 25 age and sex matched controls were included to assess to value of strain elastography as an imaging modality in detection of BACM.

Our patient and control groups had same number of patients, mostly boys consistent with the predilection of the disease to boys. There was no statistically significant difference in age, height, weight, or body mass index between the two groups (Table 1).

Although all patients had flu-like symptoms for a less than a week, the symptom which was alarming for the families and thus the reason for emergency care admission was sudden onset of inability to walk in all. In 64% of the patients this was within 24 hours after the onset of musculoskeletal symptoms.

Sonoelastography scores of the patient group differed significantly from those of the control group (p <0.05) (Table 3). The possibility of assessing the degree of tissue elasticity by elastography brings extra information, useful for diagnosis of the dynamic structural changes in the involved muscles. We believe that the increase of muscle strain ratio values is mainly caused by myositis, thus sonoelastography can also be helpful in the follow-up process of the disease.

While no statistically significant difference could be detected between the elastography scores of right and left sides either in the patient or control groups (p < 0.05 for both)-also consistent with the results from the study by Brandenburg et al. in 2015 [24], which found no significant difference between the sides tested in their pediatric study group-the final scores (the highest score obtained from right or left side) showed a statistically significant difference between the patient and control groups, mean values 4.16 +/− 0.75 versus 3.08 +/–0.40.., respectively ( p < 0.001).

This finding is different than what was reported by Güngör and Güngör [15]. Their sonoelastography findings for 32 children (16 patients diagnosed with BCAM and 16 controls) showed that gastrocnemius strain ratio values were slightly higher in the patient group when compared to controls, but this was not found to be statistically significant. Our study has a larger group of subjects consisting of 50 children (25 patients and 25 controls), and the increase in the number of patients (and controls) might have strengthened statistical accuracy.

There have been previous studies which aimed to detect active myositis in adult and pediatric population in patients with chronic diseases. These have yielded contradictory results on adults and children. In 2010 Botar-Jid et al. [10] reported decreased US elasticity of the muscles of the arms and legs in a majority of their subjects consisting of 24 adults with polymyositis and dermatomyositis and active myositis (age range 24–67 years, mean 54.8 years). In contrast, Berko et al. [14]. in 2015 demonstrated that US elastography performed poorly when used to assess for active myositis in their study in children with juvenile idiopathic inflammatory myopathy. Subjects with active myositis detected on MR imaging exhibited both normal muscle elasticity as well as decreased muscle elasticity on US elastography, while subjects with normal MRI and no evidence of active myositis also demonstrated both normal and decreased muscle elasticity, thus, revealing a low sensitivity and specificity for sonoelastography in diagnosing active myositis in this patient group.

Although there was not a consensus on the diagnostic value, there is a consensus that, on the basis of chronic inflammatory disease, muscle elasticity is either normal or decreased. However, in our series when a previously healthy child has acute myositis secondary to BACM, elastography scores are either increased or normal. We believe this is valuable information for the differential diagnosis of BACM and acute exacerbation of a chronic pathology.

In our study, when elastography scores of 4 and 5 are accepted as pathological, the sensitivity of the exam is 80%, positive predictive value 87%, specificity 88%, negative predictive value 81.5%, and overall accuracy 84 %. These are compatible with the findings from musculoskeletal MR imaging. In their series, Berko et al. [14] reported a sensitivity of 67% and specificity of 100% for MR imaging in the detection of myositis. Positive and negative predictive values were reported to be 100% and 55%, respectively.

In this study, there was discordance between the CK values and elastography scores during the course of the disease. While elastography scores tended to decrease by time, there was a nonsignificant tendency to increase for serum CK levels. We believe that elastography, which can demonstrate the status of edema and pathological changes in the muscles, has a better correlation with the decrease in patient’s complaints and symptomatology as in most cases of myositis present 48–72 h after the start of flu, and symptoms typically resolve within one week but occasionally may last up to one month [25]. Therefore, elastography follow-up for the course of the disease seems to be more reliable and can be a preferable option to serum CK levels.

The major limitation of the previous elastography studies on children with myositis was the limited number of subjects. In 2015 Berko et al.’s [14] study group included 18 children with the diagnosis of JIIM, and, recently, in 2019, Güngör et Güngör [15] studied 16 children with BACM and 16 controls. In comparison to the previous studies, our patient number consisting of 50 children totally, 25 patients with the diagnosis of BACM and the same number of controls, is the largest population studied to date. We believe that this is the the power of our statistical analysis; however, further studies should be planned on a larger scale.

Elastography is technically a challenging exam in terms of the proper application of the technique; thus, it is operator dependent [17–20]. To diminish any inconveniences due to application of elastography and any interapplicator discordance, we assigned one experienced pediatric radiologist (18 years in pediatric radiology) with the experience of sonoelastography, to carry out all the exams. Lastly, although we used an elastography scoring system to standardize the sonoelastography results, these elastography measurements are not truly quantitative and are considered subjective in nature; therefore, this also should be considered as a major limitation of this study. To increase the accuracy of this subjective evaluation, two pediatric radiologists reviewed all the data together and reached a final score for each case in consensus.

This study offers interesting information, which may constitute basis for further investigation. The efficacy of sonoelastography in differential diagnosis of myositis from neuropathies like Guillain-Barré syndrome and myopathies like Duchenne Muscular Dystrophy remains to be an area to be discovered. Again, we believe it would be interesting to investigate if elastography has any potential to replace or at least complement EMG findings in the diagnosis of these entities.

Sonoelastography combines the portability, low cost, and short study time of sonography with the ability to assess tissue elasticity. It proves to be a valuable tool in addition to US findings - in children with the diagnosis of BACM. Moreover, it is one of the available US techniques in an emergency department and may particularly evolve to become a useful routine ancillary technique for investigation and follow-up of myositis in these cases.

## Figures and Tables

**Table 1:** Elastography scoring according to color code.

Score	Color Code	Tissue Elasticity
**1**	Red predominant	Most elastic (softest)
**2**	Red with green	Mostly soft
**3**	Green predominant	Medium elastic
**4**	Green with blue	Mostly hard
**5**	Blue predominant	Least elastic (hardest)

**Table 2:** Demographic characteristics of the patient and control groups.

Demographic data	Patient group (N = 25)	Control group( N = 25)	p value

**Age (years)**			>0.05
**Range**	4.30–15.08	4.04–15.24	
**Mean +/− SD**	8.03 ± 2.84	8.11 ± 3.01	

**Height**	130.36 ± 17.27	130 ±18.99	>0.05

**Weight**	31.32 ± 11.72	32.50 ± 15.04	>0.05

**BMI**	17.88 ± 2.83	17.90 ± 3.52	>0.05

**Table 3:** Summary of elastography scores.

Elastography score (Mean ± SD)	Patient group	Control group	p value
**Right**	3.88 ± 0.83	2.76 ± 0.52	**p < 0.05**
**Left**	3.68 ± 0.75	3.00 ± 0.50	**p < 0.05**
**Final**	4.16 ± 0.75	3.08 ± 0.40.	**p < 0.05**
